# Supplementary material for: Isolation and characterization of microsatellite markers for Sturnira parvidens and cross-species amplification in Sturnira species
Source: PeerJ. 2017 May 24;5:e3367. doi: 10.7717/peerj.3367 (PMC5445947; doi:10.7717/peerj.3367)

**Supplementary material 1:** Tissues supplemented by different Museums to probe cross-species amplification and library enrichment. We are presenting museum; voucher, tissue or collector number; species and specific use of the tissue. Acronyms of museums are. MZFC (Museo de Zoología de la Facultad de Ciencias), MZUCR (Museo de Zoología de la Universidad de Costa Rica), FMNH (Florida Museum of Natural History), TTU (Texas Tech University), and LSUMZ (Lousiana State University Museum of Zoology).

| **Museum** | **Sample** | **Species** | **Utility** |
| --- | --- | --- | --- |
| MZFC-M | GHC396 | *S. hondurensis* | Cross amplification |
| MZFC-M | TACM073 | *S. hondurensis* | Cross amplification |
| MZFC-M | MRM166 | *S. hondurensis* | Cross amplification |
| MZUCR | TUCR18 | *S. burtonlimi* | Cross amplification |
| MZUCR | TUCR20 | *S. burtonlimi* | Cross amplification |
| MZUCR | TUCR48 | *S. burtonlimi* | Cross amplification |
| FMNH | 174845 | *S. oporaphilum* | Cross amplification |
| MZUCR | TUCR27 | *S. mordax* | Cross amplification |
| MZUCR | TUCR29 | *S. mordax* | Cross amplification |
| FMNH | 174871 | *S. tildae* | Cross amplification |
| FMNH | 174803 | *S erythromos* | Cross amplification |
| FMNH | 128785 | *S. bogotensis* | Cross amplification |
| FMNH | 174833 | *S. magna* | Cross amplification |
| TTU | TK104211 | *S.* new species 3 | Cross amplification |
| TTU | TK104337 | *S.* new species 3 | Cross amplification |
| TTU | TK104349 | *S.* new *species* 3 | Cross amplification |
| LSUMZ | LSUMZ393 | *S. luisi* | Cross amplification |
| LSUMZ | LSUMZ394 | *S. luisi* | Cross amplification |
| LSUMZ | LSUMZ529 | *S. luisi* | Cross amplification |
| TTU | TK56609 | *S. lilium* | Cross amplification |
| TTU | TK56808 | *S. lilium* | Cross amplification |
| TTU | TK56950 | *S. lilium* | Cross amplification |
| TTU | TK104623 | *S. bakeri* | Cross amplification |
| TTU | TK104662 | *S. bakeri* | Cross amplification |
| TTU | TK136986 | *S. parvidens* | Amplification |
| MZFC-M | GHC009 | *S. parvidens* | Illumina paired-end sequencing and amplification |
| MZFC-M | GHC032 | *S. parvidens* | Amplification |
| MZFC-M | GHC060 | *S. parvidens* | Illumina paired-end sequencing and amplification |
| MZFC-M | GHC072 | *S. parvidens* | Illumina paired-end sequencing and amplification |
| MZFC-M | GHC103 | *S. parvidens* | Amplification |
| MZFC-M | GHC213 | *S. parvidens* | Amplification |
| MZFC-M | GHC214 | *S. parvidens* | Amplification |
| MZFC-M | GHC240 | *S. parvidens* | Illumina paired-end sequencing and amplification |
| MZFC-M | GHC445 | *S. parvidens* | Amplification |
| MZFC-M | MAG011 | *S. parvidens* | Amplification |
| MZFC-M | MAG014 | *S. parvidens* | Amplification |
| MZFC-M | MCyT053 | *S. parvidens* | Amplification |
| MZFC-M | MCHAM079 | *S. parvidens* | Amplification |
| MZFC-M | MRM075 | *S. parvidens* | Illumina paired-end sequencing and amplification |
| MZFC-M | DOR013 | *S. parvidens* | Illumina paired-end sequencing and amplification |
| MZFC-M | 151CAS | *S. parvidens* | Amplification |
| MZFC-M | 210HRP | *S. parvidens* | Amplification |
| MZFC-M | MBB012 | *S. parvidens* | Amplification |
| MZFC-M | MCP079 | *S. parvidens* | Amplification |

**Supplementary material 2:** Allelic frequencies of amplified microsatellite loci**,** colors represent the absolute frequencies of the different alleles.


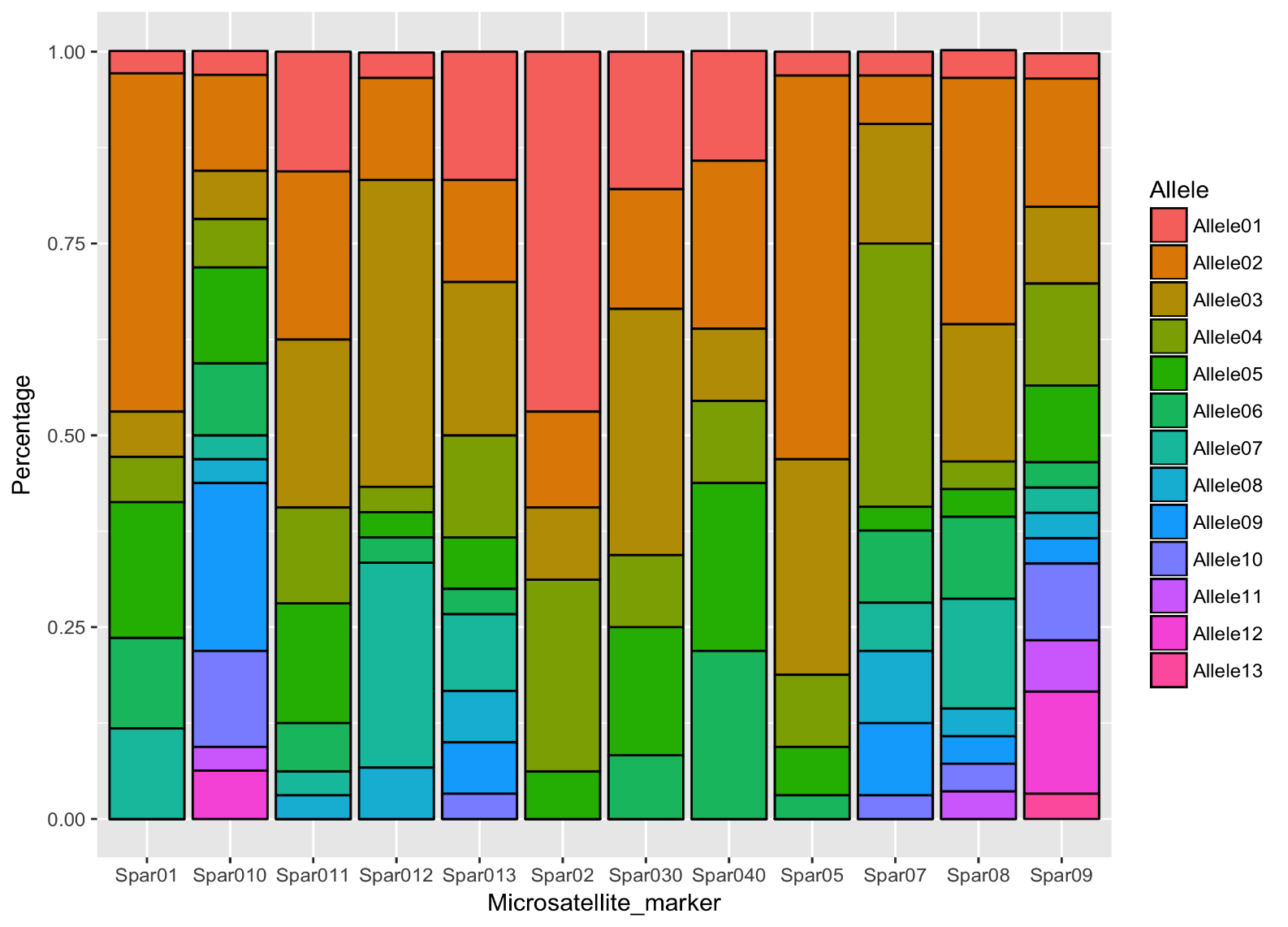

Supplement: Supplemental Information 1 [file peerj-05-3367-s001.docx]
