# Supplementary material for: Isolation and characterization of microsatellite markers for Sturnira parvidens and cross-species amplification in Sturnira species
Source: PeerJ. 2017 May 24;5:e3367. doi: 10.7717/peerj.3367 (PMC5445947; doi:10.7717/peerj.3367)
Supplement: Supplemental Information 3 [file peerj-05-3367-s003.docx]

Spar01

TGCCCTGAAGAACTTTGAGCAAAGAAAGAAAGAAAGAAAGAAAGAAAGAAAGAAAGAAAGAAAGAAAGAAAGAAAGAAAG

AAAGAAAGAAAGAAAGAAAGAAAGAAAGAAAGAAAGAAAGAAAGAAAGAAAGAAAGAAAGAAAGAAAGAAAGAAAGAAAG

AAAGAAAGAAAGAAAGAAAGAAAGAAAGAAAGAAAGAAAGAAAGAAAGAAAGAAAGAAAGAAAGAAAGAAAGAAAGAAAG

AAAGAAAGAAAGAAAGAAAGAAAGAAAGAAAGAAAGAAAGAAAGAAAGAAAGAAAGAAAGAAAGAAAGAAAGAAAGAAAG

AAAGAAAGAAAGAAAGAAAGAAAGAAAGAAAGAAAGAAAGAAAGAAAGAAAGAAAGAAAGAAAGAAAGCCCATACTTCTC

CCTCACAGC

Spar02

AGAAAGAAAGGGAGGGCGGAAAGAAAGAAAGAAAGAAAGAAAGAAAGAAAGAAAGAAAGAAAGAAAGAAAGAAAGAAAG

AAAGAAAGAAAGAAAGAAAGAAAGAAAGAAAGAAAGAAAGAAAGAAAGAAAGAAAGAAAGAAAGAAAGAAAGAAAGAAAG

AAAGAAAGAAAGAAAGAAAGAAAGAAAGAAAGAAAGAAAGAAAGAAAGAAAGAAAGAAAGAAAGAAAGAAAGAAAGAAAG

AAAGAAAGAAAGAAAGAAAGAAAGAAAGAAAGAAAGAAAGAAAGAAAGAAAGAAAGAAAGAAAGAAAGAAAGAAAGAAAG

AAAGAAAGAAAGAAAGAAAGAAAGAAAGAAAGAAAGAAAGAAAGAAAGAAAGAAAGAAAGAAAGAAAGAAAGAAAGAAAG

AAAGAAAGAAAGAAAGAAAGAAAGAAAGAAAGAAAGTTCTTTATGCCCTTTGCTCTAGG

Spar05

TGCCTGCCTAGTCTGTCACCATCATCATCATCATCATCATCATCATCATCATCATCATCATCATCATCATCATCATC

ATCATCATCATCATCATCATCATCATCATCATCATCATCATCAAGCAGTTCCCATCACATGC

Spar06

CCTGGGATGAAGTTTCTGACGTTCTTCTTCTTCTTCTTCTTCTTCTTCTTCTTCTTCTTCTTCTTCTTCTTCTTCTTC

TTCTTCTTCTTCTTCTTCTTCTTCTTCTTCTTCGAATAATGGGAATACCAGAATAAGACG

Spar07

CTCCCACGGACAATCAACGTGCTGCTGCTGCTGCTGCTGCTGCTGCTGCTGCTGCTGCTGCTGCTGCTGCTGCTGCTGC

TGCTGCTGCTGCTGCTGCTGCTGCTGCTGCCCCAGATTGCTGCCTCTCC

Spar08

GGAGTCTCCTTCATTAAGTGCCATTATTATTATTATTATTATTATTATTATTATTATTATTATTATTATTATTATTATT

ATTATTATTATTATTATTATTATTATTATTATTGGATGTGTTGTGAAGATTGTGC

Spar09

AAGTCCATTTCAAGGCTGGGACACACACACACACACACACACACACACACACACACACACACACACACACACACACACAC

ACACACACACACACACACACACACACACCCCATCATACCCTCCTTTGC

Spar010

TCTGGCCTGAGGTATTTGGGACACACACACACACACACACACACACACACACACACACACACACACACACACACACACAC

ACACACACACACACACACACACACACACACTGTAGCCACTTCCCTGCC

Spar011

AAGCCACTGCCTTGTGCCTCTCTCTCTCTCTCTCTCTCTCTCTCTCTCTCTCTCTCTCTCTCTCTCTCTCTCTCTCTCTC

TCTCTCTCTCTCTCTCTCTCTCTCTCGACTCTCTGGACATTGGCCC

Spar012

GGGAGTGAATGAGAAAGATAAAGTCCACACACACACACACACACACACACACACACACACACACACACACACACACACAC

ACACACACACACACACACACACACACACACACACCTGTCATTGCATGGGTTGG

Spar013

AAAGATTCCTGGAGATCATACCCACACACACACACACACACACACACACACACACACACACACACACACACACACACACAC

ACACACACACACACACACACACACACTGAATGTATCCTAGGGCGAGC

Spar014

TTTCTCTCACTGTCTAACTCTGCCTCTCTCTCTCTCTCTCTCTCTCTCTCTCTCTCTCTCTCTCTCTCTCTCTCTCTCTCT

CTCTCTCTCTCTCTCTCTCTCTCTCTCAGTCCTGGCAGGTGTGTCC

Spar030

AATGGCACCATATTATTCTACATAGGATTATTATTATTATTATTATTATTATTATTATTATTATTATTATTATTATTATT

ATTATTATTATTATTATTATTATTATTATTATTATTATTATTATTATTATTATTCCGTTCTAGGCTCAGTTTCC

Spar040

GACTGAGACAATTGCTTGAGATAGCATCATCATCATCATCATCATCATCATCATCATCATCATCATCATCATCATCATC

ATCATCATCATCATCATCATCATCATCATCATCATCATCATCATCGAGTTTCAGGGAGTATTTCAGTGC
